# *r*-2,*c*-6-Bis(4-fluoro­phen­yl)-*t*-3,*t*-5-dimethyl­piperidin-4-one

**DOI:** 10.1107/S1600536807068699

**Published:** 2008-01-11

**Authors:** D. Gayathri, D. Velmurugan, G. Aridoss, S. Kabilan, K. Ravikumar

**Affiliations:** a Centre of Advanced Study in Crystallography and Biophysics, University of Madras, Guindy Campus, Chennai 600 025, India; b Department of Chemistry, Annamalai University, Annamalai Nagar 608 002, India; c Laboratory of X-ray Crystallography, Indian Institute of Chemical Technology, Hyderabad 500 007, India

## Abstract

In the title compound, C_19_H_19_F_2_NO, the piperidinone ring adopts a chair conformation. The crystal packing is stabilized by C—H⋯O and C—H⋯F inter­molecular inter­actions, generating centrosymmetric dimers of *R*_2_^2^(14) and *R*_2_^2^(24) rings.

## Related literature

For related literature, see: Allen *et al.* (1987 ▶); Cremer & Pople (1975 ▶); Ganellin & Spickett (1965 ▶); Nardelli (1983 ▶); Noller & Baliah (1948 ▶).
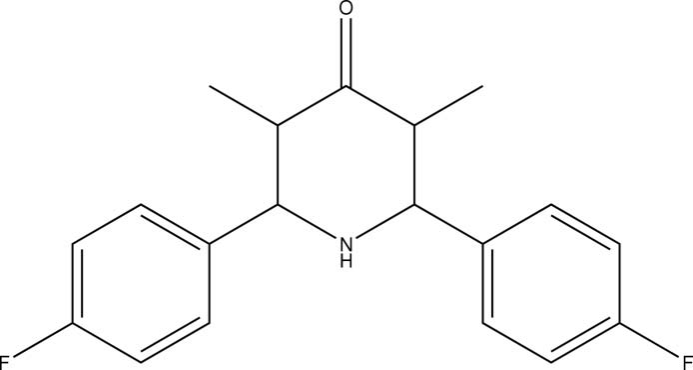


## Experimental

### 

#### Crystal data

C_19_H_19_F_2_NO
*M* *_r_* = 315.35
Monoclinic, 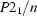

*a* = 7.3830 (6) Å
*b* = 24.0102 (19) Å
*c* = 9.4278 (7) Å
β = 101.727 (1)°
*V* = 1636.4 (2) Å^3^
*Z* = 4
Mo *K*α radiation
μ = 0.09 mm^−1^
*T* = 293 (2) K
0.27 × 0.24 × 0.22 mm

#### Data collection

Bruker SMART APEX CCD area-detector diffractometer
Absorption correction: none
18490 measured reflections
3851 independent reflections
2773 reflections with *I* > 2σ(*I*)
*R*_int_ = 0.020

#### Refinement

*R*[*F* ^2^ > 2σ(*F* ^2^)] = 0.061
*wR*(*F* ^2^) = 0.212
*S* = 1.04
3851 reflections
210 parameters
H-atom parameters constrained
Δρ_max_ = 0.52 e Å^−3^
Δρ_min_ = −0.43 e Å^−3^

### 

Data collection: *SMART* (Bruker, 2001 ▶); cell refinement: *SAINT* (Bruker, 2001 ▶); data reduction: *SAINT*; program(s) used to solve structure: *SHELXS97* (Sheldrick, 2008 ▶); program(s) used to refine structure: *SHELXL97* (Sheldrick, 2008 ▶); molecular graphics: *PLATON* (Spek, 2003 ▶); software used to prepare material for publication: *SHELXL97* and *PARST* (Nardelli, 1995 ▶).

## Supplementary Material

Crystal structure: contains datablocks I, global. DOI: 10.1107/S1600536807068699/at2525sup1.cif

Structure factors: contains datablocks I. DOI: 10.1107/S1600536807068699/at2525Isup2.hkl

Additional supplementary materials:  crystallographic information; 3D view; checkCIF report

## Figures and Tables

**Table 1:** Hydrogen-bond geometry (Å, °)

*D*—H⋯*A*	*D*—H	H⋯*A*	*D*⋯*A*	*D*—H⋯*A*
C11—H11⋯O1^i^	0.93	2.49	3.400 (3)	165
C18—H18⋯F1^ii^	0.93	2.54	3.197 (3)	128

Symmetry codes: (i) 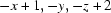; (ii) 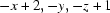.

**Fig. 1.**
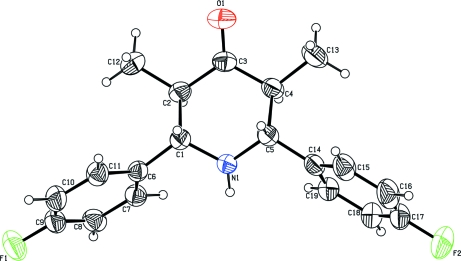
The molecular structure of title compound, showing 30% probability displacement ellipsoids.

**Fig. 2.**
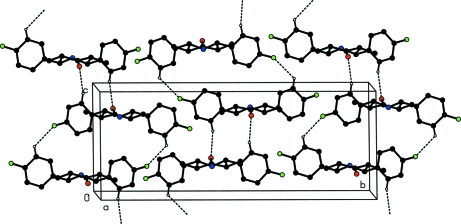
The molecular packing of (I), viewed down the a axis. For clarity, hydrogen atoms which are not involved in hydrogen bonding were omitted.

## supplementary crystallographic information

### Comment

Substituted piperidin-4-ones are important synthetic intermediates for the preparation of various alkaloids and pharmaceuticals (Ganellin and Spickett, 1965). Several substituted piperidin-4-ones and their derivatives are easily synthesized by Noller and Baliah (1948). Piperidine and their derivatives have different conformation depending on the level of substitution of heterocyclic ring. The present investigation was undertaken to establish the structure, conformation and the possible biological functions. As the substituted piperidin-4-one compounds are of great pharmaceutical importance, we have undertaken the three dimensional crystal structure determination of the title compound, by X-ray diffraction (Fig.1).

The bond lengths and bond angles are comparable with the literature values (Allen *et al.*, 1987). The flourine atoms F1 and F2 lie 0.019 (2)Å and -0.003 (2) Å, respectively, from the plane of the phenyl rings to which they are attached. The dihedral angle between the two phenyl rings is 50.4 (1)°.

The piperidinone ring adopts chair conformation with the puckering parameters (Cremer & Pople, 1975) and the smallest displacement asymmetry parameters (Nardelli, 1983) being q_2_ = 0.089 (2) Å, q_3_ = -0.573 (2) Å; Q_T_ = 0.580 (2)Å and θ = 171.2 (2)°.

The crystal packing is stabilized by C—H···O and C—H···F intermolecular interactions generating centrosymmetric dimers of *R*_2_^2^(14) and *R*_2_^2^(24) rings, respectively.

### Experimental

The title compound was prepared by the condensation of pentane-3-one, 4-flurobenzaldehyde and ammonium acetate in 1: 2: 1 molar ratio in ethanol as reported by Noller and Baliah (1948) Diffraction quality crystal was obtained by recrystalization of the crude sample from ethanol. ^1^H NMR (CDCl_3_, p.p.m): δ 0.82 (d, 6H, J=6.54 Hz), 2.73 (m, 2H), 3.59 (t, 2H, J=10.27 Hz), 2.02 (s, 1H), 7.37 and 7.03 (d, 8H).

### Refinement

All H-atoms were refined using a riding model with d(C—H) = 0.93 Å, *U*_iso_=1.2*U*_eq_ (C) for aromatic, 0.98 Å, *U*_iso_ = 1.2*U*_eq_ (C) for CH, 0.96 Å, *U*_iso_ = 1.5U _eq_ (C) for CH_3_ atoms, and with d(N—H) = 0.86 Å, *U*_iso_ = 1.2*U*_eq_ (N) for the NH group.

### Crystal data

**Table:**

C_19_H_19_F_2_NO	*F*_000_ = 664
*M**_r_* = 315.35	*D*_x_ = 1.280 Mg m^−^^3^
Monoclinic, *P*2_1_/*n*	Mo *K*α radiation λ = 0.71073 Å
Hall symbol: -P 2yn	Cell parameters from 2923 reflections
*a* = 7.3830 (6) Å	θ = 2.4–28.1º
*b* = 24.0102 (19) Å	µ = 0.09 mm^−^^1^
*c* = 9.4278 (7) Å	*T* = 293 (2) K
β = 101.7270 (10)º	Block, colourless
*V* = 1636.4 (2) Å^3^	0.27 × 0.24 × 0.22 mm
*Z* = 4	

### Data collection

**Table:**

Bruker SMART APEX CCD area-detector diffractometer	2773 reflections with *I* > 2σ(*I*)
Radiation source: fine-focus sealed tube	*R*_int_ = 0.020
Monochromator: graphite	θ_max_ = 28.1º
*T* = 293(2) K	θ_min_ = 2.4º
ω scans	*h* = −9→9
Absorption correction: none	*k* = −31→31
18490 measured reflections	*l* = −12→12
3851 independent reflections	

### Refinement

**Table:**

Refinement on *F*^2^	Secondary atom site location: difference Fourier map
Least-squares matrix: full	Hydrogen site location: inferred from neighbouring sites
*R*[*F*^2^ > 2σ(*F*^2^)] = 0.061	H-atom parameters constrained
*wR*(*F*^2^) = 0.212	*w* = 1/[σ^2^(*F*_o_^2^) + (0.1249*P*)^2^ + 0.205*P*] where *P* = (*F*_o_^2^ + 2*F*_c_^2^)/3
*S* = 1.04	(Δ/σ)_max_ < 0.001
3851 reflections	Δρ_max_ = 0.52 e Å^−^^3^
210 parameters	Δρ_min_ = −0.43 e Å^−^^3^
Primary atom site location: structure-invariant direct methods	Extinction correction: none

### Special details

**Table:**

Geometry. All e.s.d.'s (except the e.s.d. in the dihedral angle between two l.s. planes) are estimated using the full covariance matrix. The cell e.s.d.'s are taken into account individually in the estimation of e.s.d.'s in distances, angles and torsion angles; correlations between e.s.d.'s in cell parameters are only used when they are defined by crystal symmetry. An approximate (isotropic) treatment of cell e.s.d.'s is used for estimating e.s.d.'s involving l.s. planes.
Refinement. Refinement of *F*^2^ against ALL reflections. The weighted *R*-factor *wR* and goodness of fit *S* are based on *F*^2^, conventional *R*-factors *R* are based on *F*, with *F* set to zero for negative *F*^2^. The threshold expression of *F*^2^ > σ(*F*^2^) is used only for calculating *R*-factors(gt) *etc*. and is not relevant to the choice of reflections for refinement. *R*-factors based on *F*^2^ are statistically about twice as large as those based on *F*, and *R*- factors based on ALL data will be even larger.

### Fractional atomic coordinates and isotropic or equivalent isotropic displacement parameters (Å^2^)

**Table:**

	*x*	*y*	*z*	*U*_iso_*/*U*_eq_	
C1	0.6972 (2)	0.02549 (6)	0.79954 (16)	0.0546 (4)	
H1	0.7019	0.0285	0.9039	0.066*	
C2	0.4928 (2)	0.01783 (7)	0.72123 (19)	0.0624 (4)	
H2	0.4918	0.0147	0.6174	0.075*	
C3	0.3828 (2)	0.06935 (7)	0.74162 (19)	0.0637 (4)	
C4	0.4655 (2)	0.12406 (7)	0.7076 (2)	0.0637 (4)	
H4	0.4654	0.1244	0.6036	0.076*	
C5	0.6706 (2)	0.12620 (6)	0.78990 (18)	0.0585 (4)	
H5	0.6736	0.1247	0.8942	0.070*	
C6	0.8102 (2)	−0.02404 (6)	0.77288 (17)	0.0558 (4)	
C7	0.8517 (2)	−0.03400 (7)	0.63869 (18)	0.0617 (4)	
H7	0.8203	−0.0075	0.5658	0.074*	
C8	0.9388 (3)	−0.08247 (8)	0.6106 (2)	0.0734 (5)	
H8	0.9653	−0.0891	0.5197	0.088*	
C9	0.9851 (3)	−0.12059 (8)	0.7203 (3)	0.0784 (6)	
C10	0.9494 (3)	−0.11202 (8)	0.8545 (3)	0.0834 (6)	
H10	0.9829	−0.1385	0.9270	0.100*	
C11	0.8626 (3)	−0.06341 (8)	0.8813 (2)	0.0701 (5)	
H11	0.8389	−0.0569	0.9731	0.084*	
C12	0.4069 (3)	−0.03515 (10)	0.7657 (3)	0.0970 (7)	
H12A	0.4217	−0.0361	0.8692	0.145*	
H12B	0.4671	−0.0669	0.7339	0.145*	
H12C	0.2776	−0.0360	0.7220	0.145*	
C13	0.3516 (3)	0.17334 (10)	0.7391 (3)	0.1006 (8)	
H13A	0.2274	0.1698	0.6843	0.151*	
H13B	0.4054	0.2072	0.7124	0.151*	
H13C	0.3498	0.1742	0.8406	0.151*	
C14	0.7637 (2)	0.17899 (7)	0.75681 (19)	0.0638 (4)	
C15	0.8070 (3)	0.22048 (8)	0.8600 (3)	0.0826 (6)	
H15	0.7817	0.2156	0.9519	0.099*	
C16	0.8895 (4)	0.27015 (9)	0.8253 (4)	0.1013 (8)	
H16	0.9191	0.2984	0.8936	0.122*	
C17	0.9248 (3)	0.27607 (8)	0.6917 (4)	0.0966 (8)	
C18	0.8849 (3)	0.23645 (8)	0.5878 (3)	0.0898 (6)	
H18	0.9113	0.2419	0.4965	0.108*	
C19	0.8036 (3)	0.18753 (7)	0.6218 (2)	0.0720 (5)	
H19	0.7752	0.1598	0.5519	0.086*	
N1	0.76605 (17)	0.07730 (5)	0.74933 (14)	0.0547 (3)	
H1A	0.8532	0.0789	0.7012	0.066*	
O1	0.23876 (19)	0.06688 (7)	0.78279 (19)	0.0912 (5)	
F1	1.0695 (2)	−0.16840 (5)	0.6943 (2)	0.1173 (5)	
F2	1.0055 (3)	0.32416 (6)	0.6583 (3)	0.1454 (7)	

### Atomic displacement parameters (Å^2^)

**Table:**

	*U*^11^	*U*^22^	*U*^33^	*U*^12^	*U*^13^	*U*^23^
C1	0.0543 (8)	0.0562 (8)	0.0523 (8)	−0.0027 (6)	0.0080 (6)	−0.0002 (6)
C2	0.0525 (8)	0.0638 (10)	0.0695 (10)	−0.0072 (7)	0.0093 (7)	−0.0038 (7)
C3	0.0502 (8)	0.0763 (11)	0.0633 (9)	−0.0018 (7)	0.0084 (7)	−0.0059 (7)
C4	0.0509 (8)	0.0639 (10)	0.0743 (10)	0.0065 (7)	0.0079 (7)	−0.0064 (7)
C5	0.0544 (8)	0.0582 (9)	0.0605 (9)	0.0025 (6)	0.0060 (7)	−0.0076 (6)
C6	0.0510 (8)	0.0525 (8)	0.0605 (8)	−0.0040 (6)	0.0032 (6)	0.0024 (6)
C7	0.0579 (9)	0.0612 (9)	0.0634 (9)	0.0009 (7)	0.0062 (7)	0.0013 (7)
C8	0.0653 (10)	0.0724 (11)	0.0813 (12)	0.0021 (8)	0.0118 (9)	−0.0131 (9)
C9	0.0643 (11)	0.0548 (10)	0.1119 (16)	0.0029 (7)	0.0080 (10)	−0.0088 (9)
C10	0.0781 (12)	0.0635 (10)	0.1024 (15)	0.0059 (9)	0.0038 (11)	0.0227 (10)
C11	0.0735 (11)	0.0676 (10)	0.0659 (10)	0.0017 (8)	0.0068 (8)	0.0106 (8)
C12	0.0677 (12)	0.0771 (13)	0.146 (2)	−0.0166 (10)	0.0219 (13)	0.0080 (13)
C13	0.0664 (12)	0.0806 (14)	0.152 (2)	0.0176 (10)	0.0151 (12)	−0.0246 (14)
C14	0.0507 (8)	0.0528 (9)	0.0826 (11)	0.0072 (6)	0.0011 (7)	−0.0059 (7)
C15	0.0809 (13)	0.0637 (11)	0.0947 (13)	0.0047 (9)	−0.0027 (10)	−0.0171 (9)
C16	0.0944 (16)	0.0576 (11)	0.137 (2)	−0.0005 (10)	−0.0127 (15)	−0.0248 (13)
C17	0.0759 (13)	0.0525 (11)	0.155 (2)	−0.0002 (9)	0.0075 (14)	0.0060 (12)
C18	0.0858 (14)	0.0607 (11)	0.1247 (18)	0.0027 (9)	0.0259 (13)	0.0133 (11)
C19	0.0701 (10)	0.0563 (9)	0.0901 (13)	0.0003 (8)	0.0172 (9)	−0.0003 (8)
N1	0.0475 (6)	0.0518 (7)	0.0642 (8)	0.0015 (5)	0.0101 (5)	−0.0008 (5)
O1	0.0608 (8)	0.1076 (11)	0.1127 (12)	0.0004 (7)	0.0352 (8)	−0.0006 (8)
F1	0.1099 (11)	0.0692 (8)	0.1703 (15)	0.0267 (7)	0.0223 (10)	−0.0127 (8)
F2	0.1327 (14)	0.0643 (8)	0.236 (2)	−0.0278 (8)	0.0287 (13)	0.0108 (10)

### Geometric parameters (Å, °)

**Table:**

C1—N1	1.4588 (19)	C9—C10	1.359 (3)
C1—C6	1.503 (2)	C10—C11	1.379 (3)
C1—C2	1.550 (2)	C10—H10	0.9300
C1—H1	0.9800	C11—H11	0.9300
C2—C3	1.513 (2)	C12—H12A	0.9600
C2—C12	1.518 (3)	C12—H12B	0.9600
C2—H2	0.9800	C12—H12C	0.9600
C3—O1	1.206 (2)	C13—H13A	0.9600
C3—C4	1.510 (2)	C13—H13B	0.9600
C4—C13	1.516 (2)	C13—H13C	0.9600
C4—C5	1.556 (2)	C14—C19	1.379 (3)
C4—H4	0.9800	C14—C15	1.384 (3)
C5—N1	1.4595 (19)	C15—C16	1.408 (3)
C5—C14	1.504 (2)	C15—H15	0.9300
C5—H5	0.9800	C16—C17	1.345 (4)
C6—C7	1.382 (2)	C16—H16	0.9300
C6—C11	1.388 (2)	C17—C18	1.354 (4)
C7—C8	1.381 (2)	C17—F2	1.365 (3)
C7—H7	0.9300	C18—C19	1.386 (3)
C8—C9	1.371 (3)	C18—H18	0.9300
C8—H8	0.9300	C19—H19	0.9300
C9—F1	1.352 (2)	N1—H1A	0.8600
			
N1—C1—C6	112.25 (12)	C9—C10—C11	118.93 (18)
N1—C1—C2	108.44 (12)	C9—C10—H10	120.5
C6—C1—C2	110.25 (12)	C11—C10—H10	120.5
N1—C1—H1	108.6	C10—C11—C6	120.77 (18)
C6—C1—H1	108.6	C10—C11—H11	119.6
C2—C1—H1	108.6	C6—C11—H11	119.6
C3—C2—C12	112.65 (15)	C2—C12—H12A	109.5
C3—C2—C1	109.75 (13)	C2—C12—H12B	109.5
C12—C2—C1	112.88 (15)	H12A—C12—H12B	109.5
C3—C2—H2	107.1	C2—C12—H12C	109.5
C12—C2—H2	107.1	H12A—C12—H12C	109.5
C1—C2—H2	107.1	H12B—C12—H12C	109.5
O1—C3—C4	122.16 (16)	C4—C13—H13A	109.5
O1—C3—C2	122.14 (16)	C4—C13—H13B	109.5
C4—C3—C2	115.70 (13)	H13A—C13—H13B	109.5
C3—C4—C13	111.89 (16)	C4—C13—H13C	109.5
C3—C4—C5	108.50 (13)	H13A—C13—H13C	109.5
C13—C4—C5	113.54 (15)	H13B—C13—H13C	109.5
C3—C4—H4	107.6	C19—C14—C15	118.66 (18)
C13—C4—H4	107.6	C19—C14—C5	120.60 (15)
C5—C4—H4	107.6	C15—C14—C5	120.73 (18)
N1—C5—C14	111.00 (13)	C14—C15—C16	119.7 (2)
N1—C5—C4	108.39 (12)	C14—C15—H15	120.1
C14—C5—C4	111.32 (13)	C16—C15—H15	120.1
N1—C5—H5	108.7	C17—C16—C15	118.8 (2)
C14—C5—H5	108.7	C17—C16—H16	120.6
C4—C5—H5	108.7	C15—C16—H16	120.6
C7—C6—C11	118.41 (16)	C16—C17—C18	123.2 (2)
C7—C6—C1	121.60 (14)	C16—C17—F2	118.8 (2)
C11—C6—C1	119.79 (15)	C18—C17—F2	118.0 (3)
C8—C7—C6	121.34 (16)	C17—C18—C19	118.0 (2)
C8—C7—H7	119.3	C17—C18—H18	121.0
C6—C7—H7	119.3	C19—C18—H18	121.0
C9—C8—C7	118.15 (19)	C14—C19—C18	121.55 (19)
C9—C8—H8	120.9	C14—C19—H19	119.2
C7—C8—H8	120.9	C18—C19—H19	119.2
F1—C9—C10	118.7 (2)	C5—N1—C1	112.48 (13)
F1—C9—C8	118.9 (2)	C5—N1—H1A	123.8
C10—C9—C8	122.38 (18)	C1—N1—H1A	123.8
			
N1—C1—C2—C3	−53.46 (17)	C7—C8—C9—C10	−0.5 (3)
C6—C1—C2—C3	−176.73 (13)	F1—C9—C10—C11	−179.75 (17)
N1—C1—C2—C12	179.98 (15)	C8—C9—C10—C11	0.4 (3)
C6—C1—C2—C12	56.71 (19)	C9—C10—C11—C6	0.8 (3)
C12—C2—C3—O1	−3.1 (3)	C7—C6—C11—C10	−1.8 (3)
C1—C2—C3—O1	−129.78 (18)	C1—C6—C11—C10	173.17 (16)
C12—C2—C3—C4	176.70 (16)	N1—C5—C14—C19	50.64 (19)
C1—C2—C3—C4	50.01 (19)	C4—C5—C14—C19	−70.19 (19)
O1—C3—C4—C13	3.0 (3)	N1—C5—C14—C15	−130.90 (16)
C2—C3—C4—C13	−176.83 (16)	C4—C5—C14—C15	108.27 (18)
O1—C3—C4—C5	129.00 (18)	C19—C14—C15—C16	0.2 (3)
C2—C3—C4—C5	−50.79 (19)	C5—C14—C15—C16	−178.30 (17)
C3—C4—C5—N1	55.74 (18)	C14—C15—C16—C17	−0.1 (3)
C13—C4—C5—N1	−179.19 (16)	C15—C16—C17—C18	0.0 (4)
C3—C4—C5—C14	178.09 (13)	C15—C16—C17—F2	−179.76 (19)
C13—C4—C5—C14	−56.8 (2)	C16—C17—C18—C19	0.0 (4)
N1—C1—C6—C7	−50.19 (19)	F2—C17—C18—C19	179.78 (18)
C2—C1—C6—C7	70.83 (18)	C15—C14—C19—C18	−0.2 (3)
N1—C1—C6—C11	135.01 (16)	C5—C14—C19—C18	178.32 (16)
C2—C1—C6—C11	−103.97 (17)	C17—C18—C19—C14	0.1 (3)
C11—C6—C7—C8	1.7 (2)	C14—C5—N1—C1	171.33 (12)
C1—C6—C7—C8	−173.18 (15)	C4—C5—N1—C1	−66.12 (17)
C6—C7—C8—C9	−0.6 (3)	C6—C1—N1—C5	−173.32 (12)
C7—C8—C9—F1	179.65 (16)	C2—C1—N1—C5	64.62 (16)

### Hydrogen-bond geometry (Å, °)

**Table:**

*D*—H···*A*	*D*—H	H···*A*	*D*···*A*	*D*—H···*A*
C11—H11···O1^i^	0.93	2.49	3.400 (3)	165
C18—H18···F1^ii^	0.93	2.54	3.197 (3)	128

Symmetry codes: (i) −*x*+1, −*y*, −*z*+2; (ii) −*x*+2, −*y*, −*z*+1.
